# The added clinical and economic value of diagnostic testing for epilepsy surgery

**DOI:** 10.1016/j.eplepsyres.2014.02.002

**Published:** 2014-05

**Authors:** Sebastian Hinde, Marta Soares, Jane Burch, Anthony Marson, Nerys Woolacott, Stephen Palmer

**Affiliations:** a Centre for Health Economics, University of York, York, United Kingdom; b Centre for Reviews and Dissemination, University of York, York, United Kingdom; c Department of Molecular and Clinical Pharmacology, University of Liverpool, Liverpool, United Kingdom

**Keywords:** Cost-effectiveness, Added value, Seizure, Neuroimaging, Temporal lobe epilepsy, Epilepsy

## Abstract

The costs, benefits and risks associated with diagnostic imaging investigations for epilepsy surgery necessitate the identification of an optimal pathway in the pre-surgical workup. In order to assess the added value of additional investigations a full cost-effectiveness evaluation should be conducted, taking into account all of the life-time costs and benefits associated with undertaking additional investigations. This paper considers and applies the appropriate framework against which a full evaluation should be assessed.

We conducted a systematic review to evaluate the progression of the literature through this framework, finding that only isolated elements of added value have been appropriately evaluated. The results from applying the full added value framework are also presented, identifying an optimal strategy for pre-surgical evaluation for temporal lobe epilepsy surgery. Our results suggest that additional FDG–PET and invasive EEG investigations after an initially discordant MRI and video-EEG appears cost-effective, and that the value of subsequent invasive-EEGs is closely linked to the maintenance of longer-term benefits after surgery.

It is integral to the evaluation of imaging technologies in the work-up for epilepsy surgery that the impact of the use of these technologies on clinical decision-making, and on further treatment decisions, is considered fully when informing cost-effectiveness.

## Highlights

• We assess the added value of diagnostic imaging for epilepsy surgery.
• A cost-effectiveness evaluation is conducted considering a range of strategies.
• We find that additional PET and invasive EEG investigations are most cost-effective.
• The long-term effectiveness of surgery has a large impact on the optimal strategy.

## Introduction

Medical imaging represents one of the fastest growing areas of medical expenditure (Neiman Institute, 2012), this growth has been driven by both an increase in the supply of non-invasive techniques including Computed Tomography (CT), Positron Emission Tomography (PET), Single-Photon Emission Computed Tomography (SPECT) and Magnetic Resonance Imaging (MRI) technologies as well as greatly increased demand by health care providers (Iglehart, 2006). In any area of significant spend it is important to ensure that growth is based on both clinical evidence as well as value for money. Consideration of value for money ensures that additional expenditure results in the greatest potential gain in health.

However, to date there has been little research conducted, in epilepsy or more generally, that has sufficiently considered the value for money of imaging strategies available through cost-effectiveness methods. (Schaafsma et al., 2009; Burch et al., 2012a) The limited maturity of the existing research may partly be explained by the difficulty in assessing the value of an additional medical imaging technology, and lack of an accepted standard of analysis. The difficulty in assessing added value in this context is largely a result of the difficult interpretation of a test results (Hogstrom and Sverre, 1996), difficulty in interpreting and assessing diagnostic accuracy (Burch et al., 2012b), and the linking of these results to the long term clinical outcomes (Schaafsma et al., 2009; Trikalinos et al., 2009).

To date there has been little consideration of the appropriate methods with which to consider the added economic value of imaging technologies in general, with the majority focussing solely on the clinical value alone. Authors such as Schaafsma et al. (2009) and Fryback and Thornbury (1991) have presented hierarchies of evidence required to consider the added value of a diagnostic technology. A common feature of these hierarchies is that the lower end considers assessments of diagnostic performance with the higher stages considering change in clinical outcome and cost-effectiveness. In general, the evaluation of diagnostics is typically limited to the lower end of the hierarchy (Trikalinos et al., 2009). The frameworks highlight that the added value of a diagnostic technology depends not only on diagnostic accuracy but also how the results impact on subsequent treatment decisions, as well as the associated final clinical outcomes. We will consider the application of the Schaafsma hierarchy, shown in Box 1, to the example of pre-surgical workup for temporal lobe epilepsy surgery.

Surgical intervention to resect the epileptic focus has the potential to significantly improve patient outcomes (NICE, 2012). Medical imaging technologies are increasingly used to try to identify structural or functional changes to help localise the likely site of the seizure focus and inform decisions about further investigation and whether and how to proceed with surgery. Due to the costs and potential adverse events associated with these medical imaging techniques the optimal selection and order of the imaging tests is important. A systematic review of the literature found that no research had sufficiently considered the added value of imaging technologies in the pre-surgical workup of epilepsy patients, using cost-effectiveness methods (Burch et al., 2012a).

An additional review was conducted to evaluate previous research into the clinical value of tests (stage 2 of the Schaafsma hierarchy) in this area (Burch et al., 2012a) The review found a single study, by Uijl et al. (2007), which assessed the impact of additional investigations (FDG–PET and subsequent invasive-EEG) on the decision to proceed to surgery after discordant video-EEG and MRI findings. The study considered the short-term outcome following surgery (stages 1, 2 and 3); costs and longer-term outcomes were not considered.

This paper uses the study by Uijl et al. in a worked example to present a framework for assessing the full added value of additional imaging tests in the case of pre-surgical workup for temporal lobe epilepsy surgery, using the Schaafsma framework (Box 1). The framework will be used to evaluate the cost-effectiveness of the range of clinical strategies presented by Uijl et al., the uncertainty in these results will be explored through the use of a scenario analysis as well as probabilistic sensitivity analysis (PSA).

## Methods

A decision model was constructed to allow an evaluation of added value in the pre-surgical workup of patient with epilepsy consistent with the Schaafsma framework (Box 1). A decision model is a quantitative approach used to combine evidence from a variety of sources to inform the evaluation of added value. It does so through the consideration of the diagnostics outcomes of the tests alongside the longer term implications of the range of decision strategies. This approach facilitates an assessment of the relative value of each strategy available to the decision maker, in terms of costs and health related quality of life of the patient, and ultimately allows the optimal strategy to be identified through a consideration of the cost-effectiveness of each strategy. Uncertainty in the model inputs can be propagated to allow for a consideration of the likelihood and implications of an incorrect decision being made by the decision maker (Drummond et al., 2005).

This case study considers the optimal medical imaging strategy for patients considered eligible for temporal lobe epilepsy surgery, the population evaluated in the Uijl study. The decision model focuses on the appropriate course of action given discordant findings of initial MRI and EEG, and specifically assesses the added clinical and economic value of undertaking further imaging.

The decision problem addressed is limited to three possible medical investigation strategies, given non-localising or discordant findings from initial video-EEG and MRI scans, such that the clinician does not deem there to be sufficient information to proceed with surgery or to discount it. The strategies are: (1) no further imaging tests applied, (2) the use of FDG–PET alone, or (3) the use of FDG–PET followed by invasive EEG (iEEG) for patients for whom the decision to proceed to surgery is still unclear after FDG–PET. We acknowledge that these strategies represent a potential over-simplification of the available clinical pathways in both the range of technologies available (for example magnetoencephalography or the more sensitive methods such as 3T scanning), and the potential for repetition of some tests to facilitate a better understanding of the seizure focus. The strategies used in the decision model were chosen due to the lack of literature beyond that of Uijl that sufficiently considers the role of added value, and as such represent the only strategies of which sufficient information was available to facilitate inclusion into a decision model. These alternative approaches to patient management are used to define a series of separate strategies, detailed in Box 2.

The decision model developed has two main components: a short-term element, which characterises the period over which these localisation strategies are applied and, if appropriate, surgery is conducted, and a long-term element, which considers the costs and outcomes over the remaining lifetime of the patient. In developing and populating the model, we sought to incorporate evidence and treatment recommendations from the most recent NICE clinical guideline on epilepsy (NICE, 2012).

**Fig. 1:**
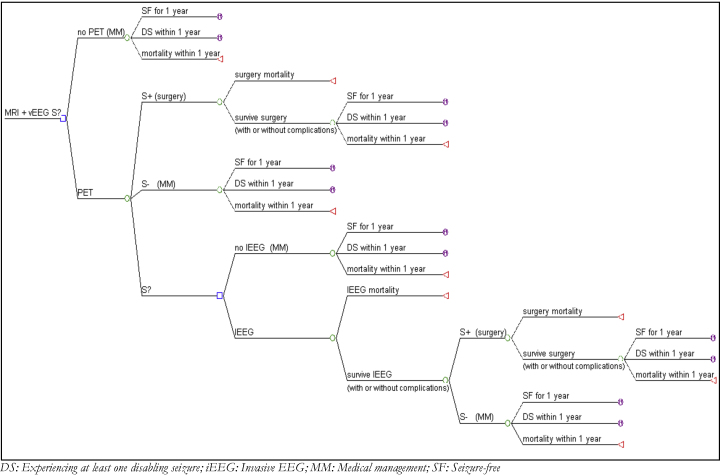
Structure of the short-term decision tree. DS: experiencing at least one disabling seizure; iEEG: invasive EEG; MM: medical management; SF: seizure-free.

The short-term element reflects the short-term clinical outcomes and adverse events associated with each of three localisation strategies, as informed by Uijl et al. (2007). For modelling purposes this is represented as a decision tree, as depicted in Fig. 1 below, and assumes a time-span of one year. The decision tree evaluates the strategies described in Box 2, such that patients are first considered in the model after having an MRI and video-EEG that has not led to a conclusive decision about eligibility for surgery. Patients flow through the model, their path determined by a set of clinical decisions (represented by *decision nodes*) and transition probabilities (*probability nodes*). At the end of the decision tree all patients have either died or enter the long-term model. Further assumptions were necessary to apply the results of Uijl et al. and are discussed elsewhere (Burch et al., 2012a).

The short-term model links decisions to undergo surgery with the immediate outcomes: those who survive surgery can either (i) achieve seizure freedom (SF) for a year after the treatment option was provided, or (ii) have a disabling seizure (DS) within that year. Our analysis uses the term ‘disabling seizure’ to represent a patient in Engel class 2 or higher, consistent with the literature used to inform the decision model (Choi et al., 2008). Patients who had seizures that were not disabling (and as such would be classified as Engel class 1) were included in the SF states. All patients were assumed to receive anti-epileptic drugs (AED) treatment in the short-term with full compliance assumed for all procedures.

**Fig. 2:**
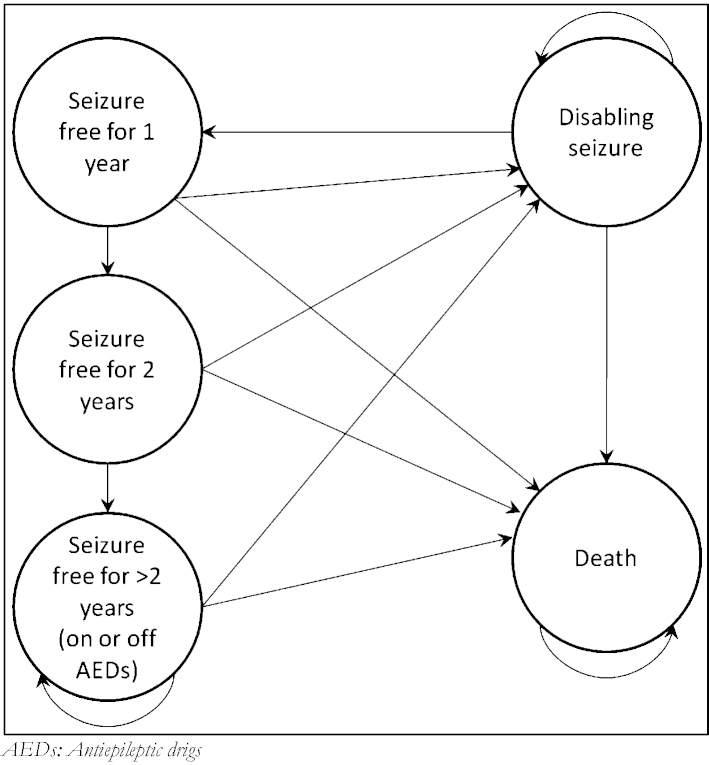
Structure of the long-term Markov model. AEDs: antiepileptic drugs.

The long-term model aims to characterise the lifetime prognosis for patients leaving the short-term model, its structure is informed by Choi et al. (2008) (see Fig. 2 below for the model structure). Patients enter directly from the short-term model having either achieved SF for the first year after evaluation or having a DS within that year, excluding those patients who died during the short-term model.

Our base case model assumes that after patients are assessed for suitability of TLE surgery, and have surgery or remain on medical management alone, they face a long term possibility of remaining seizure free dependant on the treatment received. The chance of remaining seizure free is stratified into three time periods from the time of assessment to represent the variable nature of the probability of remission identified in much of the literature (Schwartz et al., 2006; Hemb et al., 2013). These periods are the first year after assessment/surgery, from the end of the first year to the start of the fifth year, and from the fifth year to the end of the patient's lifetime. The values are based of the random-effects models estimated by Choi et al. (2008) from five studies all of which estimated the long term probability of relapse in both the surgery and medical management group.

Patients who are long-term SF (having more than two years of seizure freedom) have the possibility of stopping treatment with AEDs (with a certain probability), as is consistent with NICE clinical guidelines (NICE, 2008). From all states in the long-term model patients face the risk of death, with the risk greater for patients who have had a disabling seizure in that year than those who are seizure-free.

To inform a decision model data is required on a range of parameters which can be considered in three broad groups: (i) transition probabilities, (ii) resource use and costs and (iii) quality of life. Appendix A and B Table A1 presents the values assigned in the model to the relevant inputs, alongside the confidence intervals and distributions used to represent uncertainty in the parameter estimates. Further details are provided in the full technical report. (Burch et al., 2012a) Uncertainty in the decision model inputs is investigated through the use of PSA, which characterises the uncertainty in each of the parameters and propagates it through the decision model.

Resource use and costs are estimated from the perspective of the NHS and Personal Social Services, expressed in UK pounds sterling at a 2010 price base. Health outcomes are expressed in terms of Quality Adjusted Life Years (QALYs), a measure combining health related quality of life with duration of life. Both costs and outcomes are discounted using a 3.5% annual discount rate consistent with current guidelines (NICE, 2008).

### Scenario analysis

An important factor in the decision model is the propensity of patients who have surgery to remain seizure-free in the long-term. As discussed above our base-case analysis uses the findings from Choi's synthesis to inform the long-term probability of patients remaining seizure-free after surgery. To explore the role of long-term seizure freedom on the results of the cost-effectiveness analysis, a highly conservative scenario analysis was constructed in which the benefits of surgery were maintained for one year only after the procedure, at which point the probability of maintaining seizure freedom is assumed to be the same as patients who did not receive the surgery. This scenario represents a conservative alternative to the base-case; however it is used to highlight the importance of surgery efficacy in the evaluation of the cost-effectiveness of medical imaging strategies considered. It is therefore a useful tool in highlighting the role of the assessment of long term clinical outcomes, as represented by level 3 of the Schaafsma structure (Box 1). The results of various other scenarios are reported in the accompanying technical report (Burch et al., 2012a).

## Results

We present the results of the analysis in a number of ways, beginning with an assessment of the cost-effectiveness of the strategies considered. We then consider the implications of the uncertainty associated with the decision model both in terms of parameter uncertainty as well as the scenario analysis discussed above.

Table 1 presents the estimated mean total costs, life years and QALYs per patient derived from the decision model. The strategies themselves are ranked in terms of ascending costs and associated differences in costs and QALYs are also presented relative to the next lowest cost strategy in the table. These values are calculated from the decision model presented in the previous section. The results of the main analysis show that the lifetime costs to the health care provider associated with the three scenarios were between £23,775 and £27,696. The majority of this cost difference is made up by the cost of the additional surgical procedures arising from the additional tests provided in Strategies 2 and 3 as well as the costs of the tests themselves.

The quality of life and expected survival of patients considered under each treatment strategy, represented in QALYs over the patient's lifetime, is between 12.88 and 14.91, largely driven by the relative benefit to patients who are, as a result of outcomes from the medical imaging tests, provided with surgery.

In additon, in Table 1 we present the Incremental Cost-Effectiveness Ratios (ICER). The ICER represents the additional cost per additional QALY gained with a more costly and effective strategy, relative to the next most cost-effective alternative. The ICER is calculated as:

$$\text{ICER}=\frac{\text{incremental}\;\text{cost}}{\text{incremental}\;\text{QALY}}$$

NICE define a cost-effectiveness threshold, below which a technology is considered cost-effective, of £20,000–£30,000 per QALY (NICE, 2008). The results in Table 1 show that, at this threshold range, Strategy 3 is the most cost-effective imaging strategy, as it represents the strategy with the greatest gain in QALYs within the cost-effectiveness threshold.

We also present the probability of each strategy being cost-effective at two different threshold values (£20,000 and £30,000, Table 1). This probability is calculated using PSA methods to take account of uncertainty in many of the decision model parameters. These results show that in our main analysis Strategy 3 (i.e. the use of FDG–PET and invasive EEG) is the most likely to be cost-effective (probabilities of 0.83 and 0.84).

As discussed previously, an additional scenario was also considered whereby the benefits associated with surgery were assumed to be maintained for only one year after the procedure, in contrast to the main analysis in which these benefits were assumed to be maintained over the patient's lifetime. The results based on this scenario are shown in the lower half of Table 1.

The results of the scenario analysis show a relatively small change in the associated costs of each strategy, as a result of an increase in the number of patients expected to receive AEDs in the long term. In addition, Strategies 2 and 3 are associated with a reduction in the estimated life year (and as a result quality of life) benefits compared to the main analysis. This is as a direct result of fewer benefits being realised by those patients who have surgery, due to increased probability of future disabling seizures.

Under this scenario the cost-effectiveness of Strategies 2 and 3 become less favourable (shown by the increased ICERs). Furthermore, at both cost-effectiveness threshold values Strategy 3 is no longer cost-effective, as its ICER of £32,876 is above both thresholds. Consequently, in this scenario, Strategy 2 appears the most cost-effective strategy. However, the error probability associated with this strategy is 63–64%, and as such there exists significant uncertainty concerning the optimal strategy.

To further understand these results Table 2 provides a range of descriptive statistics from the decision model, considering both the main analysis and the scenario. The table presents the prevalence of surgery, the likelihood of patient becoming seizure-free after treatment and the average long term period of seizure freedom for each of the strategies considered.

In the base case decision model, as expected, the probability of having surgery increases as more tests are available to the clinical decision maker. These range from 0 in Strategy 1, where there is insufficient information to proceed to surgery but no further tests available, to 0.68 for Strategy 3, where both FDG–PET and invasive-EEG tests are available. The probability of being seizure-free at the end of the first year of analysis is strongly linked to the proportion of patients that receive surgical intervention; thus Strategy 3 results in the largest potential for short term seizure freedom. As the scenario analysis does not impact on the short-term decision tree the probability of having surgery and the probability of being seizure-free at the end of the short-term model (i.e. 1 year after clinical initiation) are the same in the scenario as in the base case.

The overall time a patient will spend in the seizure-free state is, alongside mortality, the main factor that will influence the QALY gain associated with each strategy and hence the relative cost-effectiveness of the strategies. Seizure-free time is, however, strongly dependent on the assumption of continued medical benefit of surgery, as tested by the scenario, where the duration of benefit is reduced from lifetime to one year only. As no patients in Strategy 1 receive surgery the duration is unchanged in the scenario. In contrast, Strategies 2 and 3 the expected seizure-free durations are significantly reduced by this change in assumption (by 8.04 and 9.76 years, respectively). While the seizure-free duration is significantly reduced the relative rank is unchanged, with Strategy 3 still expected to result in the longest period of seizure freedom.

## Discussion

This paper has sought to address the limitations in the existing literature addressing added value of imaging technologies by considering the cost-effectiveness of pre-surgical workup strategies related to FDG–PET. The model developed was based on the best available clinical and economic evidence identified at the time of funding using systematic reviews. The results of the main analysis found that the use of FDG–PET for those patients in whom the decision to proceed to surgery was unclear following video EEG and routine MRI, followed by invasive EEG for patients with indeterminate FDG–PET results (Strategy 3) appeared the most cost-effective strategy at conventional cost-effectiveness thresholds of £20,000 to £30,000 per QALY. However, when the benefits of surgery were assumed to last for only one year, as represented by the scenario, the additional value of using invasive EEG as a final test after FDG–PET became less apparent. Since the completion of this research further studies have been published relating to the long term clinical evidence around seizure freedom (Edelvik et al., 2013; Hemb et al., 2013), while these studies have not been included in the model their findings are consistent with the modelled approach, strengthening the findings of our analysis.

We consider that the results of this analysis are important in several respects. First, they provide a clear indication that some form of non-invasive testing after an initially discordant MRI and video-EEG appears cost-effective, and that the value of subsequent invasive-EEGs is closely linked to the maintenance of benefits after surgery. Second, as discussed by Gazelle et al. (2005) the model demonstrates that the added value of the visualisation strategies is inextricably linked both to their impact on the decision to proceed to surgery and the cost-effectiveness of the subsequent treatments.

Our research has, additionally, highlighted significant limitations in the literature around the pre-surgical workup for epilepsy surgery, with only one paper deemed to have appropriately collected and reported the value to the decision maker of each additional medical investigation. (Uijl et al., 2007) Our paper, therefore, provides an important stepping stone from the previous work conducted by authors such as Uijl et al. (2005), Burch et al. (2012b) in the evaluation of added value of imaging technologies in the pre-surgical workup of epilepsy surgery. However, the analysis represents a simplification of the large range of imaging modalities available to clinicians, such as magnetoencephalography scans, and is unable to consider the role of factors such as the use of repeated MRIs, a direct result of the lack of literature accurately considering added value of additional investigations to the clinician.

Finally our analysis makes the important link in the existing stages of the evidence hierarchy by constructing a robust decision model able to assess the added value of a medical imaging technology through the use of cost-effectiveness evaluation. This is the natural development of previous studies assessing the clinical added value of a visualisation test. (Burch et al., 2012b) As such the findings of this research are of relevance to areas beyond this case study, having demonstrated the proper and complete application of the framework for analysis laid out by Fryback and Thornbury (1991), Schaafsma et al. (2009).

## Disclosure

None of the authors has any conflict of interest to disclose.

## Funding

This report was funded by the NIHR HTA Programme (project number HTA 09/106/01) and will be published in full in Health Technology Assessment; see the HTA website for further details of this project (http://www.hta.ac.uk). The views and opinions expressed therein are those of the authors and do not necessarily reflect those of the HTA Programme, NIHR, NHS, or the Department of Health. Any errors are the responsibility of the authors.

## Figures and Tables

**Table 1:** Cost-effectiveness results (base-case versus alternative scenario), see Box 2 for Strategy definitions.

Strategy	Cost (£)	Life years	QALYs incremental Cost (£)		Incremental QALYs	ICER (£)	Probability of being most cost-effective	
							£20,000	£30,000
Main analysis								
Strategy 1—MM	23,775	18.78	12.88	–	–	–	0.14	0.13
Strategy 2—PET	26,621	19.80	14.58	2846	1.70	1679	0.03	0.03
Strategy 3—PET and iEEG	27,696	20.01	14.91	1075	0.33	3227	0.83	0.84
Alternative scenario (no long-term benefits of surgery)								
Strategy 1—MM	23,726	18.78	12.89	–	–	–	0.36	0.25
Strategy 2—PET	27,207	18.84	13.19	3482	0.30	11,526	0.37	0.36
Strategy 3—PET and iEEG	28,416	18.84	13.23	1208	0.04	32,876	0.27	0.39

**Table 2:** Descriptive results (base-case and alternative scenario).

Strategy	Probability of having surgery	Probability of being seizure-free at year 1	Overall time SF state, years
Main analysis			
Strategy 1	0.00	0.08	2.35
Strategy 2	0.56	0.44	11.82
Strategy 3	0.68	0.52	13.85
Alternative scenario (no long-term benefits of surgery)			
Strategy 1	0.00	0.08	2.35
Strategy 2	0.56	0.44	3.78
Strategy 3	0.68	0.52	4.09

## Box 1 The Schaafsma framework

1. The first step of evaluation is to identify the *test characteristics* of the imaging technology. These are the parameters that define the clinical ability of the technology, for example the diagnostic accuracy, and the sensitivity and specificity.
2. The second considers the *clinical value* of a test. As the tests involved in pre-treatment workup are usually used in sequence the additional information provided by the test to the decision maker must be considered, rather than the test's ability in isolation.
3. Thirdly the resultant *clinical outcome* is important to the evaluation of the technology being considered. As the justification for additional imaging tests is not the direct output of the test but the potential access to beneficial medical treatments, such as surgery, it is vital to consider the potential clinical outcomes of the range of different treatment options available once the test result is defined.
4. Finally is the role of *cost-effectiveness*. Schaafsma argues that the only suitable method to combine all of the information collected in the previous stages is the use of a full cost-effectiveness evaluation.

## Box 2 Strategies evaluated

Strategy 1)—MM No additional tests performed, and all patients receive medical management (MM).

Strategy 2)—PET FDG–PET is performed; if the result of this test does not lead to a positive (S+) or negative (S−) decision to undertake surgery and still leaves the clinician uncertain whether to proceed to surgery (S?), then no further tests are performed. The analyses consider:

- Patients are offered surgery if S+ after FDG–PET.
- Patients are offered medical management if S− or S? after FDG–PET.

Strategy 3)—PET and iEEG FDG–PET is performed; if the result of this test does not lead to a clear decision on whether the patient should proceed to surgery (S?), iEEG is offered. The analyses consider:

- Patients are offered surgery if S+ after FDG–PET.
- Patients are offered medical management if S- after FDG–PET.
- Patients are offered invasive EEG if S? after FDG–PET. The result of this test determines the provision of treatment (S+ after invasive EEG implies a decision to proceed to surgery, whilst S− and S? assume medical management).
