# Supplementary material for: The added clinical and economic value of diagnostic testing for epilepsy surgery
Source: Epilepsy Res. 2014 May;108(4):775–81. doi: 10.1016/j.eplepsyres.2014.02.002 (PMC4000270; doi:10.1016/j.eplepsyres.2014.02.002)
Supplement: Supplementary file 1 [file mmc1.docx]

**Appendices (to be submitted as Supporting Information)**

**Appendix 1: Decision Model Inputs**

**Table A1:** Parameter values used in the decision model

| **Parameter** | **Base case value (95% CI)** | **Distribution** | **Source** |
| --- | --- | --- | --- |
| **Short term model** | | | |
| Probability of S+ after FDG-PET | 0.56 (0.46, 0.65) | Beta | *Uijl et al. (2007)* |
| Probability of S- after FDG-PET | 0.20 (0.12, 0.29) | Beta |  |
| Probability of S? after FDG-PET | 0.14 (0.08, 0.22) | Beta |  |
| Probability of S+ after iEEG | 0.83 (0.74, 0.89) | Beta | *Uijl et al. (2007)* |
| Probability of S- after iEEG | 0.17 (0.10, 0.25) | Beta |  |
| Probability of transient complications, Surgery/iEEG | 0.08 (0.06, 0.1) | Beta | *Choi et al. (2008)* |
| Probability of permanent complications, Surgery/iEEG | 0.04 (0.02, 0.06) | Beta |  |
| Probability of mortality after Surgery or iEEG | 0.003 (0, 0.0075) | Beta |  |
| TP of SF patients in the 1st year, MM | 0.08 (0, 0.16) | Beta | *Choi et al. (2008)* |
| TP of SF patients in the 1st year, Surgery | 0.719 (0.695, 0.743) | Beta |  |
| **Long term model** |  |  |  |
| TP from SF to DS after the 1st year, MM | 0.254 (0.109, 0.462) | Beta | *Choi et al. (2008)* |
| TP from DS to SF between 1st and 5th years, MM | 0.047 (0.03, 0.07) | Beta |  |
| TP from DS to SF after the 5th year, MM | 0.016 (0.01, 0.023) | Beta |  |
| TP from SF to DS between 1st and 5th years, Surgery | 0.056 (0.029, 0.083) | Beta | *Choi et al. (2008)* |
| TP from SF to DS after the 5th year, Surgery | 0.042 (0.016, 0.068) | Beta |  |
| TP from DS to SF between 1st and 5th years, Surgery | 0.059 (0.09, 0.11) | Beta |  |
| TP from DS to SF after the 5th year, Surgery | 0.02 (0.002, 0.072) | Beta |  |
| For patients in SF | 1.11(0.63, 1.93) | Log normal | *Choi et al. (2008)* |
| For patients in DS, MM | 5.64 (3.49, 9.12) | Log normal |  |
| For patients in DS, Surgery | 5.42 (3.97, 7.77) | Log normal |  |
| **Costs** |  |  |  |
| FDG-PET | £398 | Fixed | *NHS reference costs 2009-10* |
| Invasive EEG | £3,306 | Fixed | *NHS reference costs 2009-10* |
| Surgery (no complications) | £6,171 | Fixed | *NHS reference costs 2009-10* |
| Surgery complication | £700 | Fixed | *NHS reference costs 2009-10* |
| Cost of an annual course of AEDs to all patients | £448.80 / 6 months | Fixed | *NICE Guideline 20 and author assumptions* |
| Non-drug costs of a seizure-free patient | £110 | Gamma | *NICE Guideline 20* |
| Non-drug costs of a seizure persistent patient | £482 | Gamma |  |
| **Health Related Quality of Life (HRQoL)** |  |  |  |
| *Disutilities in relation to general population* |  |  |  |
| for SF in MM | 0.00 (-0.08, 0.08) | Normal | *Choi et al. (2008)* |
| for SF in Surgery, no complication | 0.00 (-0.06, 0.06) | Normal |  |
| *Disutilities in relation to SF states* |  |  |  |
| for DS in MM | -0.21 ( -0.62, -0.02) | Gamma | Choi *et al.* (2008) |
| for SF in Surgery, permanent complication | -0.20 (-0.65, -0.01) | Gamma |  |
| for SF in Surgery, transient complication | -0.01 (-0.01, 0.00) | Gamma |  |
| for DS in Surgery, permanent complication | -0.31 (-0.83, -0.04) | Gamma |  |
| for DS in Surgery, transient complication | -0.22 (-0.62, -0.03) | Gamma |  |

Abbreviations: TP – Transition probability; MM – Medical management; SF- Seizure-freedom; DS: disabling seizures; MM: Medical management; SF: Seizure freedom; TP: Transition probability
